# Camera- and Viewpoint-Agnostic Evaluation of Axial Postural Abnormalities in People with Parkinson’s Disease through Augmented Human Pose Estimation

**DOI:** 10.3390/s23063193

**Published:** 2023-03-16

**Authors:** Stefano Aldegheri, Carlo Alberto Artusi, Serena Camozzi, Roberto Di Marco, Christian Geroin, Gabriele Imbalzano, Leonardo Lopiano, Michele Tinazzi, Nicola Bombieri

**Affiliations:** 1 Department of Engineering for Innovation Medicine, University of Verona, 37134 Verona, Italy; 2 Department of Neuroscience “Rita Levi Montalcini”, University of Turin, 10124 Turin, Italy; 3 Neurology 2 Unit, Azienda Ospedaliero-Universitaria Città della Salute e della Scienza di Torino, 10126 Turin, Italy; 4 Neurology Unit, Movement Disorders Division, Department of Neurosciences Biomedicine and Movement Sciences, University of Verona, 37129 Verona, Italy

**Keywords:** axial postural abnormalities, human pose estimation, deep learning, Parkinson’s disease

## Abstract

Axial postural abnormalities (aPA) are common features of Parkinson’s disease (PD) and manifest in over 20% of patients during the course of the disease. aPA form a spectrum of functional trunk misalignment, ranging from a typical Parkinsonian stooped posture to progressively greater degrees of spine deviation. Current research has not yet led to a sufficient understanding of pathophysiology and management of aPA in PD, partially due to lack of agreement on validated, user-friendly, automatic tools for measuring and analysing the differences in the degree of aPA, according to patients’ therapeutic conditions and tasks. In this context, human pose estimation (HPE) software based on deep learning could be a valid support as it automatically extrapolates spatial coordinates of the human skeleton keypoints from images or videos. Nevertheless, standard HPE platforms have two limitations that prevent their adoption in such a clinical practice. First, standard HPE keypoints are inconsistent with the keypoints needed to assess aPA (degrees and fulcrum). Second, aPA assessment either requires advanced RGB-D sensors or, when based on the processing of RGB images, they are most likely sensitive to the adopted camera and to the scene (e.g., sensor–subject distance, lighting, background–subject clothing contrast). This article presents a software that augments the human skeleton extrapolated by state-of-the-art HPE software from RGB pictures with exact bone points for posture evaluation through computer vision post-processing primitives. This article shows the software robustness and accuracy on the processing of 76 RGB images with different resolutions and sensor–subject distances from 55 PD patients with different degrees of anterior and lateral trunk flexion.

## 1. Introduction

Parkinson’s disease (PD) is the second most common neurodegenerative disease and is characterized by non-motor and motor symptoms [1,2,3,4,5,6]. Among the latter, axial postural abnormalities (aPA) are a frequent complication associated with back pain, reduced mobility and postural instability, thus leading to higher risk of falls and reduced quality of life [7,8,9]. Clear definitions and cut-off values for axial postural abnormalities in people with PD and atypical Parkinsonisms were recently given to avoid heterogeneity of the reported results and lack of clarity in the literature, and to foster advances on diagnosis, management and prevention [10].

Among aPA, camptocormia (CC) and Pisa syndrome (PS) indicate reversible severe flexions of the trunk on the sagittal (with thoracic fulcrum—tCC: anterior flexion at C7–T12 vertebrae $>45^{\circ }$; with lumbar fulcrum—lCC: anterior flexion at L1–L5 vertebrae $>30^{\circ }$ and hip flexion) and coronal planes (lateral flexion $>10^{\circ }$), respectively, [10]. Their reliable evaluation and early recognition may help tuning pharmacological [11] and physical [12] therapies for their management [13].

The analysis of posture is normally performed with stereophotogrammetric systems, force platforms [14,15] and inertial sensors [16]. To the best of the authors’ knowledge, these approaches are not normally used to assess CC and PS. The only exception is given in [17], where the authors proposed a marker-based approach complemented with a ground reaction force analysis to investigate the effects of PS on standing posture and gait symmetry, with a particular focus on joint kinematics and weight distribution. However, no direct assessment of aPA was proposed.

aPA have been recently evaluated with Kinovea [18]. Kinovea is an open-source video annotation tool able to measure angles after virtual palpation of landmarks on RGB images/videos (www.kinovea.org, accessed on 2 September 2022). Following an equivalent approach, NeuroPostureApp© (www.neuroimaging.uni-kiel.de, accessed on 2 September 2022) provides clinicians with PS and CC measures, both with the lumbar and thoracic fulcrum [19].

The Task Force on Postural Abnormalities in Parkinsonism, within the International Movement Disorders Society, has recently established the consensus on nosology and cut-off values, and recommended the use of either the NeuroPostureApp©, or the wall goniometer [20] as state-of-the-art methods to evaluate aPA, with the wall goniometer potentially underestimating such measures [10].

The NeuroPostureApp© has been developed by Kiel University, following the definitions given in [19,21] and calls for an operator to collect a picture of the undressed subject and to virtually palpate landmarks on that picture. Then, NeuroPostureApp© can calculate the following angles: (i) tCC, defined as the external angle between the line joining the fulcrum of the spine flexion and the fifth lumbar vertebra process (L5), and the line connecting the fulcrum of the spine flexion to the seventh cervical vertebra process (C7); (ii) lCC, defined as the external angle between the line joining L5 and the visible lateral malleolus, and the line connecting L5 to C7; (iii) PS, which is the external angle between the line joining the midpoint between the feet and L5, and the line connecting L5 to C7 [19]. The intra-subject test–retest and the inter-operator reliability were found to be excellent for CC and good for PS evaluation [21]. However, virtual palpation of landmarks is strongly operator-dependent, calling for extensive training and is thus time-consuming.

Software based on human pose estimation (HPE) [22] could be a valid markerless alternative to virtual landmark recognition and palpation. HPE algorithms are based on convolutional neural networks (CNN) that automatically identify feature points of the human body, defined as keypoints, on images captured through standard digital cameras [23,24]. There is increasing interests from the scientific community in the application of HPE algorithms to study motion and posture, with many validation studies have been published [25,26].

A HPE approach has been recently used to assess aPA in people with PD [27]. Nevertheless, keypoints normally identified by HPE algorithms are not sufficient to assess aPAs as defined by the Movement Disorders Society criteria [10]. Specifically, the missing keypoints needed to measure CC with thoracic and lumbar fulcra, and PS are: the last cervical vertebra (C7), the last lumbar vertebra (L5), the mid-point between the two ankles (MA), and the most distant point on the participant silhouette from the line joining C7 and L5 on the sagittal view (FC).

Zhang et al. proposed the use of a depth camera (RGB-D sensor, Microsoft Kinect v2) to extend the standard set of HPE keypoints, detecting the human silhouette [28]. Recently, several solutions exist to extrapolate depth information both directly (i.e., through RGB and matrix depth sensors) or indirectly (i.e., stereo RGB cameras) [26]. Although being validated and reliable solutions, there is a trade-off between the usability, portability and accuracy of such systems [29]. Moreover, these methods call for specific devices to be available, or specialized users to be involved, possibly limiting their applicability to every-day use.

More recently, a software-based tool, called AutoPosturePD, was proposed to automatically evaluate PS, lCC and tCC from RGB pictures of people with PD, taken with single off-the-shelf cameras (i.e., with no need of depth information) and no additional human input (e.g., virtual landmark palpation) [30]. AutoPosturePD measures PS, lCC and tCC as defined by the Task Force on Postural Abnormalities in Parkinsonism of the International Movement Disorders Society [10]. The authors presented an agreement analysis between the measures obtained with NeuroPostureApp© (i.e., the gold standard) [19] and the newly proposed AutoPosturePD, with the aim of evaluating its accuracy in the diagnosis of aPA (reporting Bland–Altman plots, intra-class correlation coefficients, standard error of measurements and Cohen’s kappa), and encompassing sensitivity and specificity in the diagnosis of PS and CC against the current gold standard [30]. AutoPosturePD was found to be a valid tool for the clinical assessment of of PS, lCC and tCC in PD, supporting their diagnosis [30]. AutoPosturePD was used to measure the aPA of each participant and classify whether they had passed the thresholds [10] to define a pathological condition, thus running a sensitivity and specificity analysis of the software and obtaining excellent results [30].

It is worth noting that AutoPosturePD automatically measures the aPA starting from images taken with a single off-the-self camera, with no additional information to be fed to the algorithm. Parameters that could prevent AutoPosturePD from accurately identifying landmarks include the subject’s anthropometry, image resolution, the ratio between the subject image size and total image size, and the hue saturation values of the images.

However, AutoPosturePD’s robustness to participant anthropometry and picture characteristics, ensuring its portability to different devices, was not tested.

The aim of this work is to fill this gap, presenting a secondary analysis on the same dataset used in [30] and to test the robustness of AutoPosturePD outcome measures to the above-mentioned parameters, ensuring its portability to different devices and different environmental settings (i.e., viewpoints, background colours, room lighting, etc.). This article also presents a more extended and deep description of the software.

## 2. Materials and Methods

### 2.1. The AutoPosturePD Software

The state-of-the-art HPE solutions to implement inference on images (or video frames) to extrapolate a set of human body keypoints (KPS):

(1) $$\begin{array}{c} KPS=\{kp_{j}^{i}:\;i=1\ldots |VF|,\;j=1\ldots |CNN\_kps|\} \end{array}$$

where $kp_{j}^{i}$ is the *j*-th keypoint at the *i*-th frame, and |CNN_kps| is the total number of keypoints detected through the adopted convolutional neural network (CNN) per frame, with |VF| being the number of processed video frames. All HPE platforms provide a common subset of canonical keypoints (i.e., estimates of the human joint centres or segment centroid), including left and right shoulders (LSH,RSH), elbows (LE,RE), wrists (LW,RW), pelvis (*P*), knees (LK,RK), ankles (LA,RA), and face points such as nose (*N*), eyes (LEye,REye), and ears (LEar,REar).

**Figure 1:**
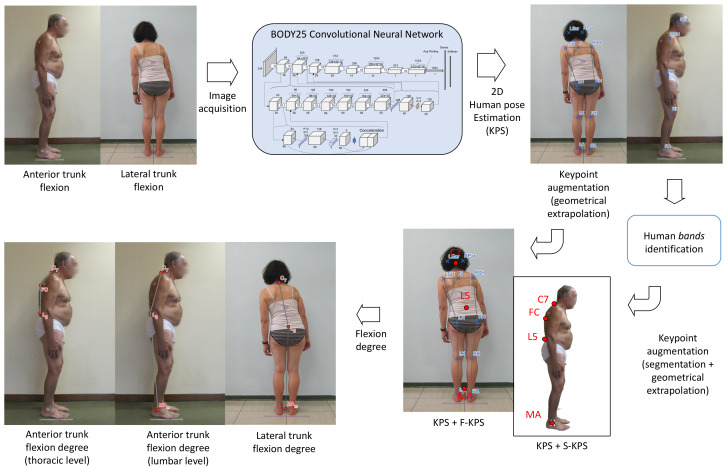
Overview of the measurement pipeline.

Figure 1 shows the platform overview used to augment the set of canonical KPS with two additional sets of keypoints, F−KPS and S−KPS as proposed in [30], for the assessment of PS, and lCC and tCC, respectively. OpenPose [31], being an accurate state-of-the-art HPE software [32], was used to extrapolate the canonical 2D human keypoints. We selected the BODY25 model trained with the COCO [33] and MPII datasets [34] to train OpenPose and extrapolate a set of 25 keypoints.

#### 2.1.1. F−KPS for Frontal View Analysis—PS Assessment

**Figure 2:**
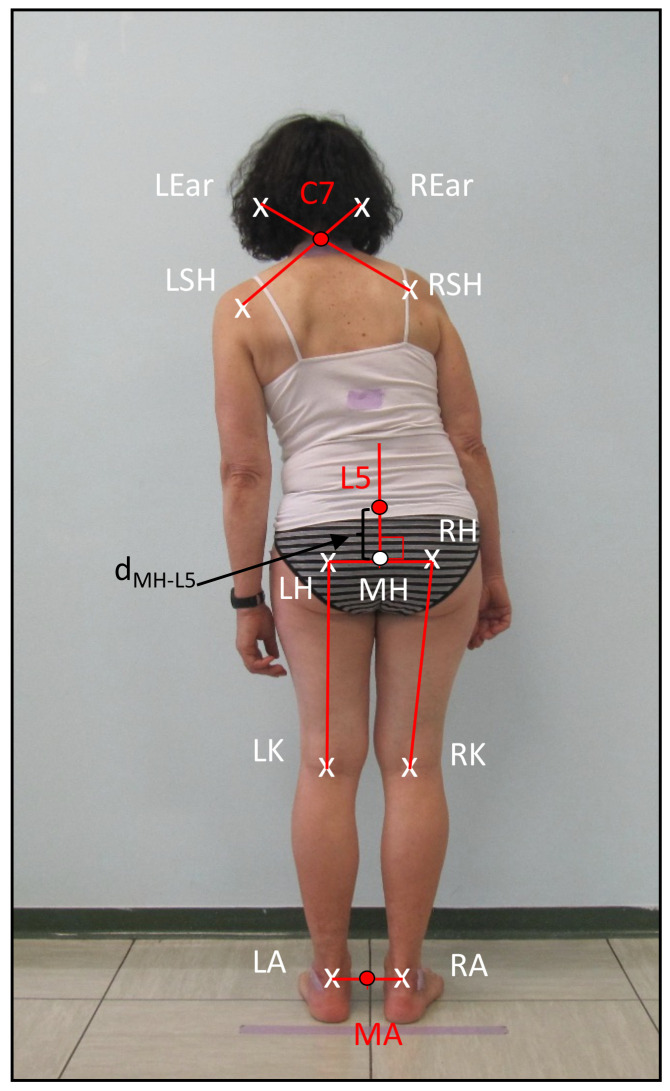
Geometrical extrapolation of F-KPS keypoints.

We defined the subset F−KPS={C7,L5,MA}, where C7, L5, and MA are the seventh cervical vertebra, the fifth lumbar vertebra and the mid-point between the two ankles, respectively. They are extrapolated geometrically from the canonical KPS as follows from a frontal plane image of the subject. C7 is the most prominent point on the sagittal view of the neck along the spine [35] (see Figure 2). AutoPosturePD geometrically identifies this point as the intersection of the segments connecting the shoulder keypoints (LSH,RSH) to the ear keypoints (LEar,REar), as shown in Figure 2. L5 corresponds to the first spinal process under an imaginary line connecting the two iliac crests [35]. AutoPosturePD first identifies the middle point (MH) between the left hip (LH) and right hip (RH). Starting from MH, it draws a vertical segment and identifies L5 at a distance $d_{MH-L5}$:

(2) $$\begin{array}{c} d_{MH-L5}=K_{1}\%\left(avg\left[\left(LH-LK\right),\left(RH-RK\right)\right]\right) \end{array}$$

This is a parametric percentage ($K_{1}=20$) of the average left and right leg length, estimated as the distance between the hip (LH and RH for the left and right side, respectively) and the knee keypoint (LK and RK for the left and right side, respectively). $K_{1}$ is a user-defined parameter that was empirically extrapolated from our experimental results to map L5 on the fifth lumbar vertebra by taking advantage of the ground truth.

Finally, AutoPosturePD identifies MA as the mid-point between the two ankle keypoints (LA,RA).

#### 2.1.2. S−KPS for Sagittal View Analysis—lCC and tCC assessment

We defined the subset S−KPS={C7,FC,L5,MA}, given on subjects’ images taken from the sagittal view, and with FC being the most distant point from the line joining *C*7 and *L*5 lying on the subject’s silhouette. The points C7 and L5 also lie on the subject’s silhouette. Different from [28], which relied on the depth information provided by RGB-D sensors (i.e., Microsoft Kinect v2), AutoPosturePD extrapolates the subject’s silhouette through the processing of the RGB pictures.

**Figure 3:**
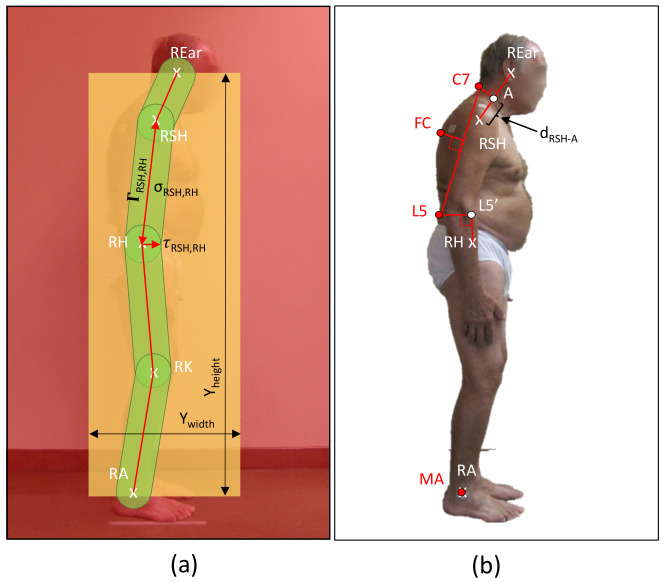
Silhouette rendering and masking (**a**) and geometrical extrapolation of S-KPS keypoints (**b**).

Matching between the subject underwear and background colours, as well as the environment lighting can strongly impact the accuracy of the subject edge extrapolation. To reduce accuracy loss, AutoPosturePD implements silhouette rendering and masking as a first step. This procedure applies a graph cut algorithm (Figure 3a) to extract three class variants [36]: certain foreground, probable background, certain background. The software iteratively processes the image and finds the best solution to map each pixel in one of the following classes:

- Red—background: pixels outside the box ($Y_{width}\times Y_{heigth}$) created around the subject, where $Y_{width}=REar_{y}-RA_{y}$. $Y_{width}$ is defined by the user. These pixels are not considered for the edge extrapolation to reduce false positive pixels.
- Green—foreground: pixels inside the bands connecting adjacent joints: ear with shoulder ($\Gamma_{REar,RSH}$), shoulder with hip ($\Gamma_{RSH,RH}$), hip with knee ($\Gamma_{RH,RK}$), knee with ankle ($\Gamma_{RKJ,RA}$); likely representing the subject’s limbs.
- Yellow—probable background: pixels are neither of the previous classes.

The foreground is identified as follows:

- the segment $\sigma_{ij}=\bar{P_{i}P_{j}}$ joining two keypoints $P_{i}$ and $P_{j}$, {i,j}=1…|CNN_kps|;
- the segment thickness $\tau_{ij}\in R_{0}^{+}$, which is upper bounded by the radius of the body segment, obtained geometrically through distances between the HPE keypoints;
- the band $\Gamma_{ij}=\left(\sigma_{ij},\tau_{ij}\right)$, defined as the area covered by $\sigma_{ij}$ when isotropically expanded by $\tau_{ij}$.

From the segmented image, AutoPosturePD extrapolates C7 as follows (see Figure 3b). It first identifies *A* in the segment REar−RSH such as:

(3) $$\begin{array}{c} d_{RSH-A}=K_{2}\%\left(REar-RSH\right) \end{array}$$

This is a parametric percentage ($K_{2}=40$) of the distance between the right ear and shoulder keypoints (REar and RSH, respectively). The $K_{2}$ was chosen as the value that empirically minimizes the error between C7 extrapolated by the software and ground truth. Starting from *A*, it identifies C7 as the last point of the mask within the line perpendicular to the segment connecting the ear and shoulder, passing via *A*.

L5 is extrapolated via two phases. AutoPosturePD extrapolates $L5^{\prime }$ with the same approach used for L5 in the frontal view. Then, starting from $L5^{\prime }$, it implements a search process to find the last segmented pixel of the subject silhouette. With the same value $K_{1}$ = 20 as for the frontal view, we empirically observed an estimation error that is negligible for most of the subjects. The software extrapolates FC starting from the segment C7−L5 and moving perpendicularly backwards to the silhouette. For the sagittal view, AutoPosturePD identifies MA as coincident with the right ankle keypoint.

### 2.2. Participants and Ethics Statement

76 pictures were collected from 55 PD outpatients from sagittal and frontal views when clinically relevant. PD participants (39 males; age: 71 ± 9 years old; BMI: 25.07 ± 3.37 kg/m^2^) were enrolled at the Neurology Unit, University Hospital of Verona (Verona, Italy), and at the Neurology 2 Unit, University-Hospital “Città della Salute e della Scienza” (Turin, Italy) [30]. More clinical and demographical data are provided in [30].

The study was approved by the Ethics Committee for clinical trials of Verona and Rovigo (protocol code 1655CESC, 14 March 2018) and all participants provided their written informed consent prior to participating in the study.

### 2.3. Procedure

Participants were asked to stand barefoot as still as possible, wearing their underwear only in front of a neutral wall (i.e., the background was not corrupted by other images or elements). Considering participants’ clinical evaluation, a total of 76 pictures were taken: 25 recording the frontal plane of participants, which were used to detect and measure PS; and 51 recording the sagittal plane of participants, which were used to detect and measure lCC and tCC [10]. Pictures were taken with the lens on level of patients’ hip and from either a strict posterior (for PS evaluation), or lateral view (for lCC and tCC evaluation) [19].

Each picture was analysed both with the NeuroPostureApp© [19], whose results were taken as the ground truth, and with AutoPosturePD.

### 2.4. Statistical Analysis

The validity of the measures obtained with the AutoPosturePD against those taken as the ground truth (i.e., those obtained with the NeuroPostureApp© [19]) were tested in [30] and are beyond the aim of the present research.

Given the described processing procedure, it is worth highlighting that: (i) images used to test and develop AutoPosturePD were taken with various off-the-shelf devices and, thus, at various resolutions and hue–saturation–variance; (ii) operators were not instructed to take pictures at specific distances from the targeted person with PD; and (iii) an intrinsic variability of participants’ anthropometry (i.e., the body mass index—BMI) could serve as an additional confusing factor for the algorithm.

To check for AutoPosturePD measure sensitivity to all these quantities, the Pearson’s correlation coefficient (*R*) and the significance of the correlation (p<0.05) were computed between the AutoPosturePD measured angle and: (i) the participants’ body mass index (BMI); (ii) the image size in pixels (width, height and area); (iii) the participants’ cover factor, obtained as the ratio between the subject image size and the total image size in pixels (width, height and area); and (iv) the colour characteristics of the analysed picture (hue–saturation—variance (HSV)).

Moreover, to also check for AutoPosturePD measurement error sensitivity to the same quantities and strengthen the validation study proposed in [30], the Pearson’s correlation coefficient (*R*) and the significance of the correlation (p<0.05) were computed between the measurement error (i.e., the difference between AutoPosturePD and NeuroPostureApp© measurements) and: (i) the participants’ body mass index (BMI); (ii) the image size in pixels (width, height and area); (iii) the participants’ cover factor, obtained as described above; and (iv) the colour characteristics of the analysed picture (HSV).

Statistical analyses were performed in RStudio (version 2022.12.0+353, Boston, MA, USA).

## 3. Results

**Figure 4:**
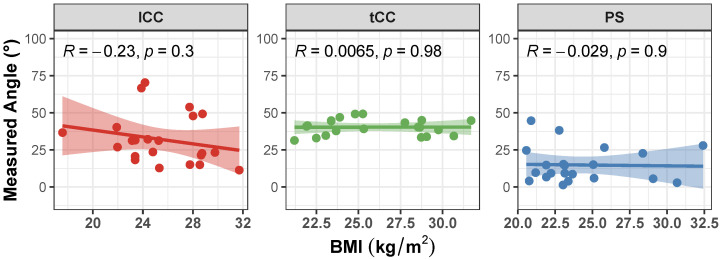
Scatter plot with Pearson’s correlation coefficient (*R*) and relevant significance (*p* value) of the AutoPosturePD measured angle against the participants’ body mass index (BMI, kg/m^2^): Anterior trunk flexion with lumbar fulcrum (lCC) in red; camptocormia with thoracic fulcrum (tCC) in green; and Pisa syndrome (PS) in blue.

**Figure 5:**
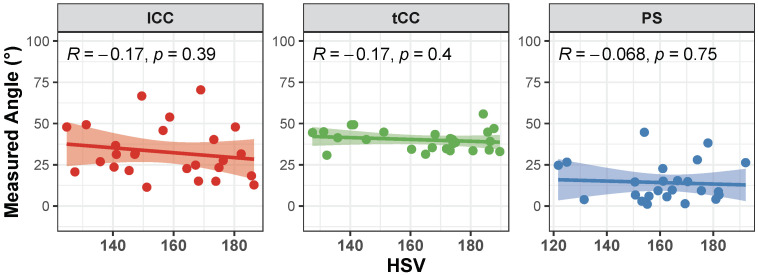
Scatter plot with Pearson’s correlation coefficient (*R*) and relevant significance (*p* value) of the AutoPosturePD measured angle against the picture hue–saturation–variance (HSV): camptocormia with lumbar fulcrum (lCC) in red; camptocormia with thoracic fulcrum (tCC) in green; and Pisa syndrome (PS) in blue.

**Figure 6:**
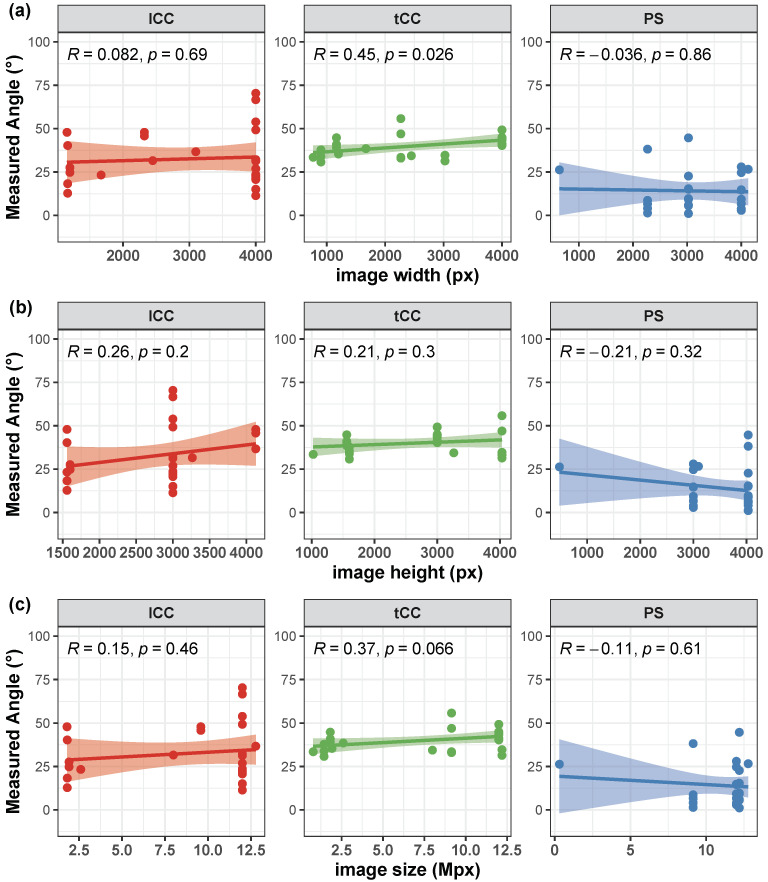
Scatter plot with Pearson’s correlation coefficient (*R*) and relevant significance (*p* value) of the AutoPosturePD measured angle against the image size (image width in panel (**a**); image height in panel (**b**); and total image area in panel (**c**)): camptocormia with lumbar fulcrum (lCC) in red; camptocormia with thoracic fulcrum (tCC) in green; and Pisa syndrome (PS) in blue.

**Figure 7:**
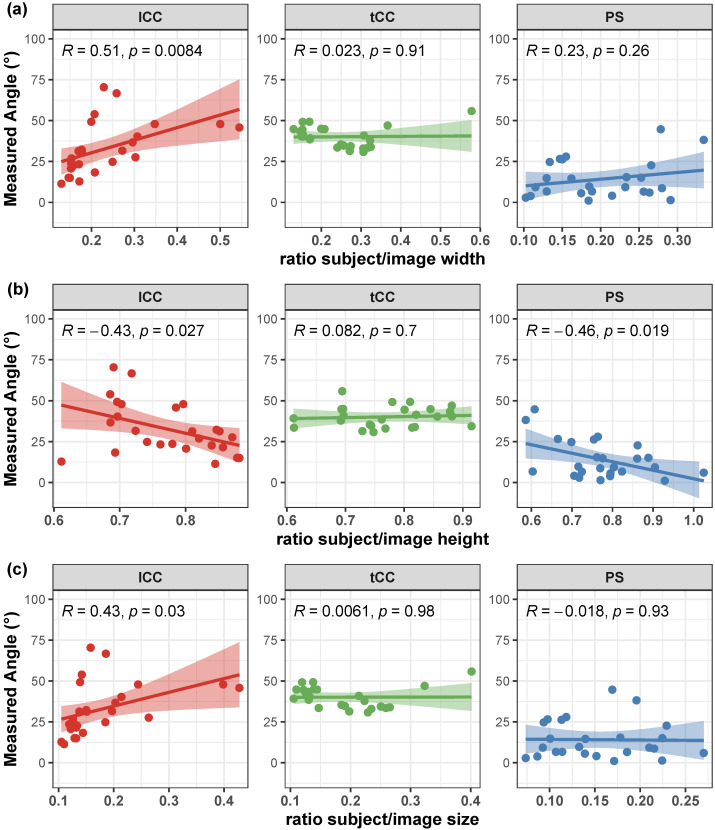
Scatter plot with Pearson’s correlation coefficient (*R*) and relevant significance (*p* value) of the AutoPosturePD measured angle against the participants’ cover factor (ratio between participants width and image width in panel (**a**); ratio between participants height and image height in panel (**b**); and ratio between total participants’ area and image area in panel (**c**)): camptocormia with lumbar fulcrum (lCC) in red; camptocormia with thoracic fulcrum (tCC) in green; and Pisa syndrome (PS) in blue.

Correlation of the AutoPosturePD measures to participants’ anthropometry were not significant considering all the aPA (−0.23<R<0.0065 and p>0.30; see Figure 4). Similar results were obtained when looking at the correlation of the measured angles with the picture characteristics: no significant correlations with colour characteristics (−0.17<R<−0.068 and p>0.39; Figure 5), and image size (−0.21<R<0.45 and p>0.066; Figure 6). Significant correlation was instead obtained for: tCC measures with respect to image width (R=0.46 and p=0.026; Figure 6a); and lCC measures with respect to width cover factor (R=0.51 and p=0.0084; Figure 7a), height cover factor (R=−0.43 and p=0.027; Figure 7b), and area cover factor (R=0.43 and p=0.03; Figure 7c).

**Figure 8:**
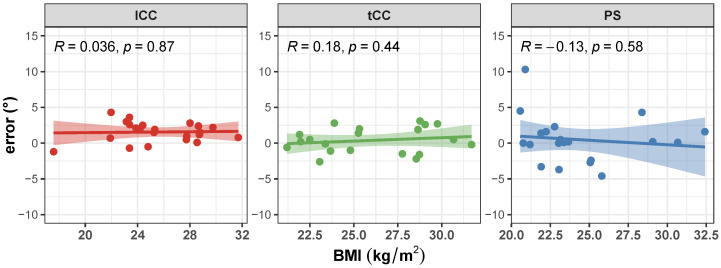
Scatter plot with Pearson’s correlation coefficient (*R*) and relevant significance (*p* value) of the error (i.e., the difference between the AutoPosturePD and the NeuroPostureApp© measurements) against the participants’ body mass index (BMI, kg/m^2^): anterior trunk flexion with lumbar fulcrum (lCC) in red; camptocormia with thoracic fulcrum (tCC) in green; and Pisa dyndrome (PS) in blue.

**Figure 9:**
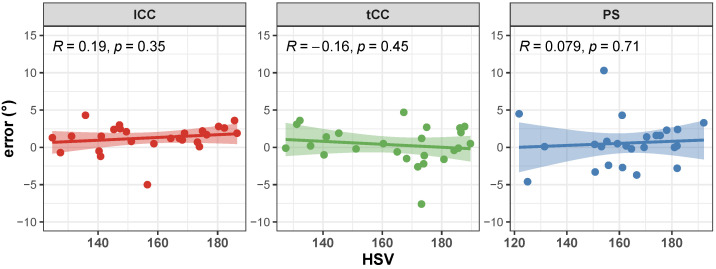
Scatter plot with Pearson’s correlation coefficient (*R*) and relevant significance (*p* value) of the error (i.e., the difference between the AutoPosturePD and the NeuroPostureApp© measurements) against the picture hue–saturation—variance (HSV): camptocormia with lumbar fulcrum (lCC) in red; camptocormia with thoracic fulcrum (tCC) in green; and Pisa syndrome (PS) in blue.

**Figure 10:**
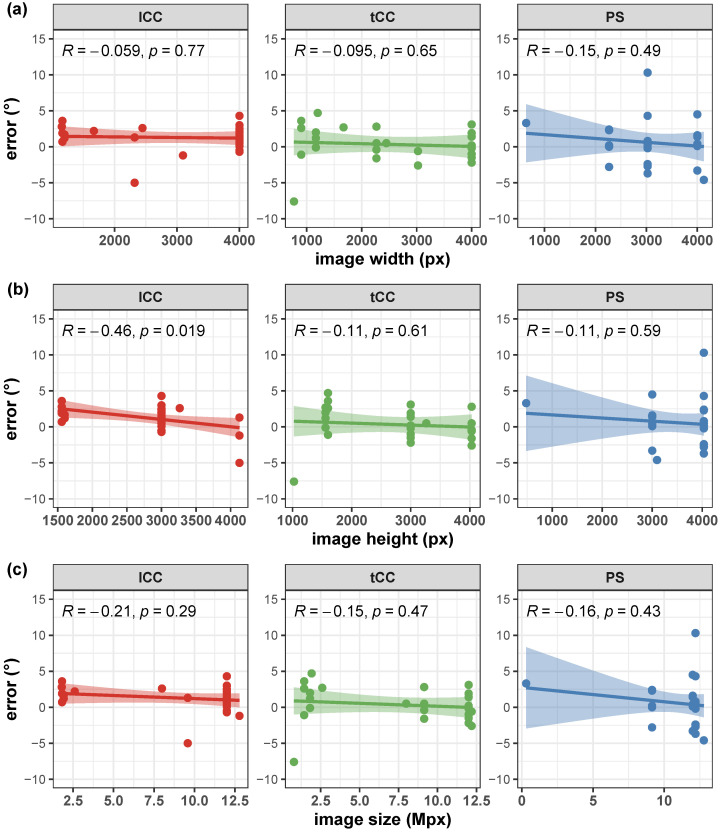
Scatter plot with Pearson’s correlation coefficient (*R*) and relevant significance (*p* value) of the error (i.e., the difference between the AutoPosturePD and the NeuroPostureApp© measurements) against the image size (image width in panel (**a**); image height in panel (**b**); and total image area in panel (**c**)): camptocormia with lumbar fulcrum (lCC) in red; camptocormia with thoracic fulcrum (tCC) in green; and Pisa syndrome (PS) in blue.

**Figure 11:**
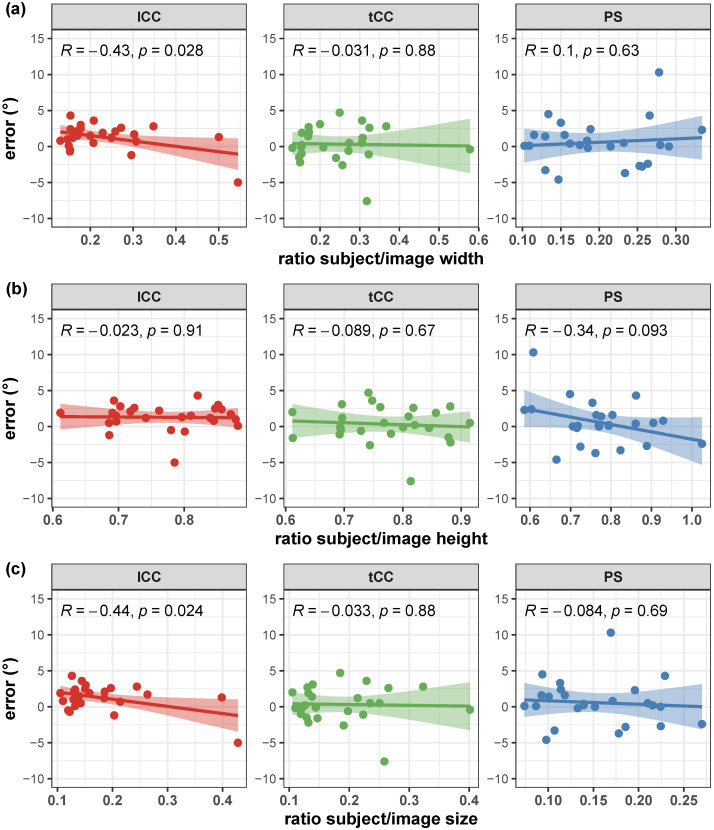
Scatter plot with Pearson’s correlation coefficient (*R*) and relevant significance (*p* value) of the error (i.e., the difference between the AutoPosturePD and the NeuroPostureApp© measurements) against the participants’ cover factor (ratio between participants’ width and image width in panel (**a**); ratio between participants’ height and image height in panel (**b**); and ratio between total participants’ area and image area in panel (**c**)): camptocormia with lumbar fulcrum (lCC) in red; camptocormia with thoracic fulcrum (tCC) in green; and Pisa syndrome (PS) in blue.

Correlation of the AutoPosturePD measurement error to participants’ anthropometry and to picture characteristics (i.e., image size, participants cover factor, and colour characteristics) were not significant considering all the aPA (−0.21<R<0.19 and p>0.29). See Figure 8, Figure 9, Figure 10 and Figure 11 for details. Exceptions were obtained for the lCC abnormality with respect to: image height (R=−0.46 and p=0.019, Figure 10b); width cover factor (R=−0.43 and p=0.028, Figure 11a); and area cover factor (R=−0.44 and p=0.024, Figure 11c).

## 4. Discussion

Early and reliable detection of axial postural abnormalities (aPA) in people with PD, such as camptocormia and Pisa syndrome, is clinically relevant for their prompt management and treatment [10]. A previous study presented a novel low-cost solution, called AutoPosturePD, for the automatic and reliable evaluation of camptocormia with lumbar and thoracic fulcrum (lCC and tCC, respectively), and Pisa syndrome (PS) [30]. The proposed algorithm automatically builds a set of keypoints through silhouette extraction [36] and geometrical post-processes images of people with PD taken with off-the-shelf RGB cameras, initially processed with a state-of-the-art HPE platform [31,32]. The strengths of AutoPosturePD are: (i) to not only consider the canonical keypoints obtained with HPE algorithms [27], which are not sufficient to estimate aPA when dealing with people with PD [10]; (ii) to not call for any external reference as in [27]; (iii) to call for 2D, rather than 3D images, as in [28], avoiding the need of specific instruments to be purchased and managed; and (iv) to be potentially used retrospectively on pictures taken beyond the clinic, avoiding patients needing to travel to clinical facilities. Moreover, the results obtained with the present study demonstrated that AutoPosturePD is robust to participants’ anthropometry (i.e., BMI) and to picture characteristics (i.e, image size, the ratio between the pixels covered by the participant and the total image picture, and hue–saturation–variance), ensuring its portability to different devices and different environmental settings (i.e., viewpoints, background colours, room lighting, etc.).

AutoPosturePD measures have been proven to be in agreement with those taken as the ground truth (i.e., those obtained with the NeuroPostureApp© [19]) and encourage the use of this novel tool to evaluate PS, lCC and tCC [30]. Indeed, correlation analysis and agreement between the two measures were very good [30], with systematic bias and the limit of agreements lower than the minimal detectable changes (MDC) for the same measures obtained with the NeuroPostureApp© ($MDC_{lCC}=3.7^{\circ }$; $MDC_{tCC}=6.7^{\circ }$; $MDC_{PS}=2.1^{\circ }$) [21], or other conventional methods (X-ray images: $MDC_{PS}=5^{\circ }$ [37]; bubble inclinometer: $MDC_{CC}=13.7^{\circ }$ [38]).

It is worth noting that the measures taken as the ground truth are strongly operator-dependent. Indeed, the operator is asked to virtually palpate a few landmarks on a picture: the fulcrum of the spine flexion (FC), the most prominent process of the fifth lumbar vertebra (*L*5), the most prominent process of the seventh cervical vertebra (*C*7), and either the lateral malleoli or the mid-point between the feet (MA; depending on the sought measure: PS or lCC and tCC, respectively). An inaccuracy in the palpation of these points, would lead to a measurement error, that could hinder both the correct quantification of the axial postural abnormality, and the worst or better performance of AutoPosturePD with respect to NeuroPostureApp©. Most likely, the AutoPosturePD approach can overcome these limitation as it is based on automatic image processing to detect the keypoints used to measure PS, lCC and tCC angles. Factors that could hamper good keypoint identification could be associated with subjects’ anthropometry and images’ characteristics (i.e., the image size, the ratio between subject and image sizes, and the colour characteristics). The presented results demonstrated that AutoPosturePD measurement error is, though, robust to all the aforementioned variables (Figure 8 and Figure 9). Significant but weak correlations were obtained for lCC measures with respect to image height (R=−0.46 and p=0.019, Figure 10b); width cover factor (R=−0.43 and p=0.028, Figure 11a); and area cover factor (R=−0.44 and p=0.024, Figure 11c). Similarly, the analysis performed on the AutoPosturePD outcomes demonstrated robustness of PS, lCC and tCC measurements to participants’ anthropometry (Figure 4) and image characteristics (Figure 4, Figure 5, Figure 6 and Figure 7). Weak to moderate correlations were instead obtained for PS with respect to subject/image height cover factor (R=−0.46 and p=0.019; Figure 7b), and for lCC measures with respect to subject/image width cover factor (−0.43<R<0.51 and p<0.03; Figure 7). These results suggest that the wider the subject image, the better aPA could be evaluated.

The presented findings lead to the conclusion that any picture taken with the RGB camera of a commercial smartphone could be sufficient to run this novel tool and still obtain reliable results on the evaluation of aPA in people with PD.

A few limitations of this study should be acknowledged. Among the 55 PD participants enrolled for the development of AutoPosturePD, 4 had both PS and tCC, 1 had PS and lCC, 12 had both tCC and lCC, and 2 had the coexistence of PS, tCC and lCC [30]. The authors also performed a sensitivity and specificity analysis on the AutoPosturePD in detecting aPA, considering those with no PS as controls for the PS classification, and those with no lCC and tCC as controls for the lCC and tCC classification, respectively, [30]. Results showed excellent classification performances of AutoPosturePD but the high heterogeneity and small sample size calls for a more substantial analysis, where a proper control group is included (i.e., participants with no diagnosed of axial postural abnormalities). This analysis, together with a repeatability and reproducibility analysis, is mandatory for the introduction of this tool into clinical practice. The repeatability analysis (i.e., the agreement of measures obtained with the same methodology applied by the same operator and device and on the same subject [39]) must be performed feeding AutoPosturePD with different pictures of the same participant taken by the same operator. A multi-centre clinical trial would also be useful to conduct a reproducibility analysis (i.e., the agreement of measures obtained with the same methodology applied by different operators and devices [39]).

The closeness and agreement of the measurements with those taken as the ground truth and obtained with the NeuroPostureApp© [19] are promising. Moreover, although having considered AutoPosturePD sensitivity to the subject/image cover factor, a proper sensitivity analysis to test the effect of camera–subject distance on outcomes (e.g., with a repeated measure design) was not performed. The lCC measurements dependence on the ratio of pixels covered by the subject and the total number of pixels (image width, height and area), despite being weak, suggests that a deeper understanding of this aspect is worth exploring, potentially leading to the implementation of a guiding frame to properly take pictures of patients with PD to evaluate sagittal axial postural deformities with AutoPosturePD.

Future works should consider designing a multi-centre clinical trial, enrolling a larger number of participants with PD with the inclusion of a appropriate control group. Considering a population of people with atypical Parkinsonisms and aPA not associated with PD would also foster AutoPosturePD validation for its use diagnosing other movement disorder medical conditions. As part of this clinical trial, the repeatability and reproducibility of measurements should be tested, together with a sensitivity analysis of AutoPosturePD to define an appropriate camera–subject distance. From the obtained results—those presented here and in previous research [30]—could lead to the development of a portable app easily available to clinicians. Moreover, the potential of this approach could be relevant to many other applications in aPA involving the spine, such as the non-invasive screening of idiopathic scoliosis.

## 5. Conclusions

AutoPosturePD is a novel low-cost software-based automatic and portable tool for the evaluation of axial postural abnormalities in people with Parkinson’s disease, which relies on the processing of images taken with off-the-shelf RGB cameras. This tool provides clinicians with reliable measurements of axial postural abnormalities, robust to differing operator expertise, image characteristics and subjects’ anthropometry. Its use could foster the diagnosis, management, and prevention of axial postural abnormalities.

## Data Availability

Data collected and used for this study are available upon reasonable request to the corresponding author.

## Abbreviations

The following abbreviations are used in this manuscript:

aPA	Axial postural abnormalities
C7	Seventh cervical vertebra
CC	Camptocormia
CNN	Convolutional neural networks
HPE	Human pose estimation
KPS	Keypoints
L5	Fifth lumbar vertebra
LA	Left ankle joint centre
lCC	Lumbar camptocormia
LE	Left elbow joint centre
LH	Left hip joint centre
LK	Left knee joint centre
LSH	Left shoulder joint centre
LW	Left wrist joint centre
MA	Mid-point of the two ankles
PD	Parkinson’s disease
PS	Pisa syndrome
RA	Right ankle joint centre
RE	Right elbow joint centre
RH	Right hip joint centre
RK	Right knee joint centre
RSH	Right shoulder joint centre
RW	Right wrist joint centre
tCC	Thoracic camptocormia
VF	Video-frames
